# Supplementary material for: Monotherapy cancer drug-blind response prediction is limited to intraclass generalization
Source: PLoS Comput Biol. 2026 Apr 22;22(4):e1013232. doi: 10.1371/journal.pcbi.1013232 (PMC13128123; doi:10.1371/journal.pcbi.1013232)
Supplement: S1 Text — (PDF) [file pcbi.1013232.s001.pdf]

Supplementary Text: Monotherapy cancer drug-blind response prediction is limited to intraclass generalization

William Herbert, Nicholas Chia, Paul Jensen, Marina Walther-Antonio

April 13, 2026

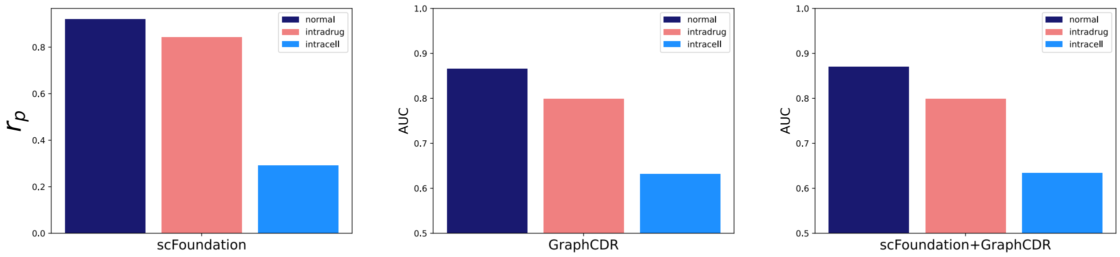

Fig A: **Intradrug permutation in advanced cell line representations and alternative model architectures does not deviate from previously observed patterns.** Permutation experiment performance for a transformer based cell line representation (scFoundation), an alternative graph neural network architecture (GraphCDR), and a combination of the two (scFoundation+GraphCDR). Advanced cell line representations and advanced architectures do not overcome the tendency of models to overfit to drug representations. GraphCDR binarizes drug response into responders/nonresponders, so results are displayed using AUC rather than Pearson correlation.

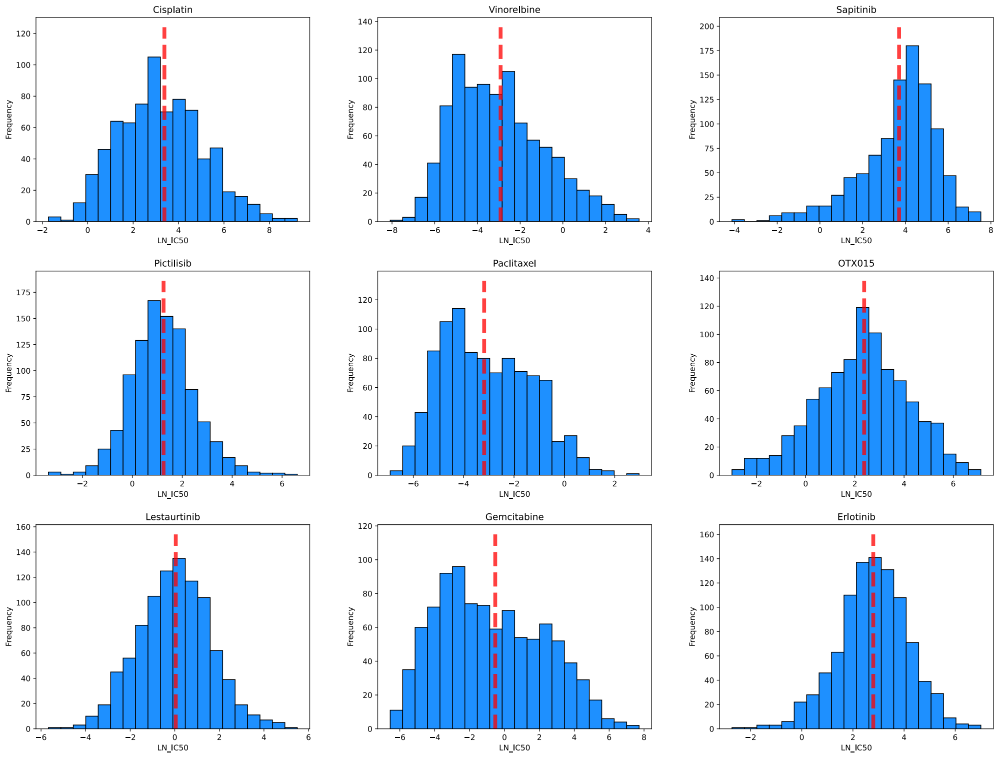

Fig B: **Intradrug permutation drives prediction of drugs' average response.** Response distribution for each drug is shown by the blue histogram. The constant prediction obtained from intradrug permutation experiments is shown by the red, vertical dashed line. The constant prediction obtained from permutation experiments corresponds to the mean response value for each drug.

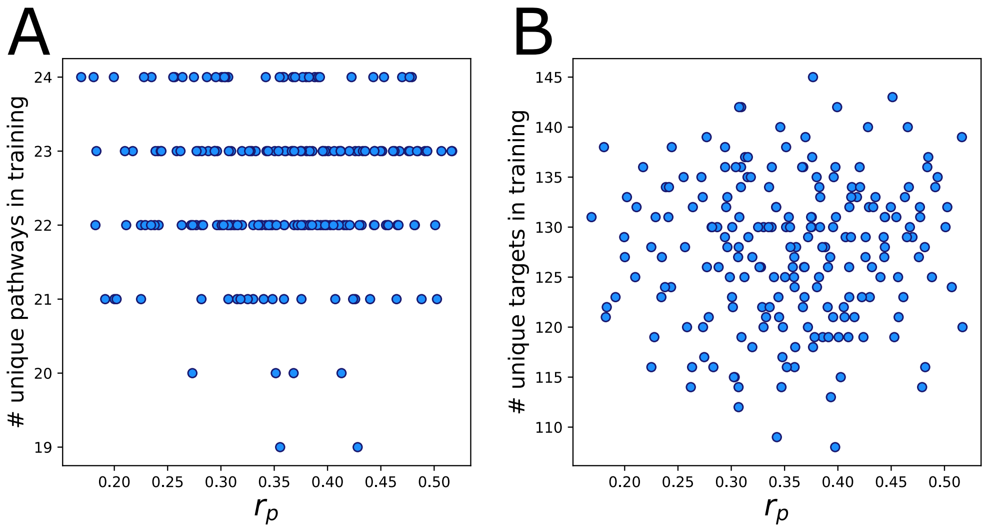

Fig C: **Training set uniqueness is not correlated with test set performance.** Training set uniqueness is measured in terms of number of unique (A) broader pathway level targets or (B) specific drug targets.

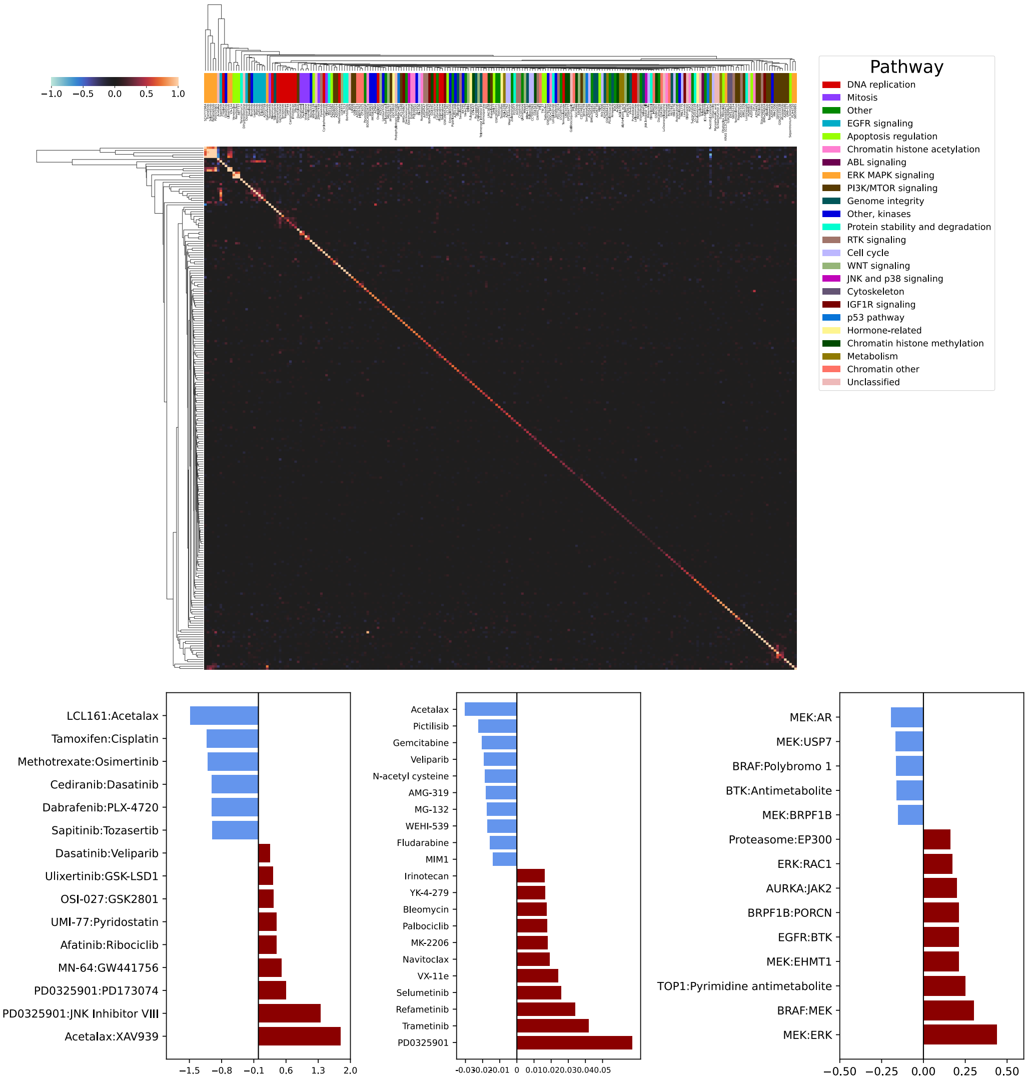

Fig D: **Coefficients of drugs present during training in a model using scFoundation embeddings.** One-to-one (bottom left), many-to-one (bottom middle), and class-to-class (bottom right) relationships are calculated from heatmap coefficients.

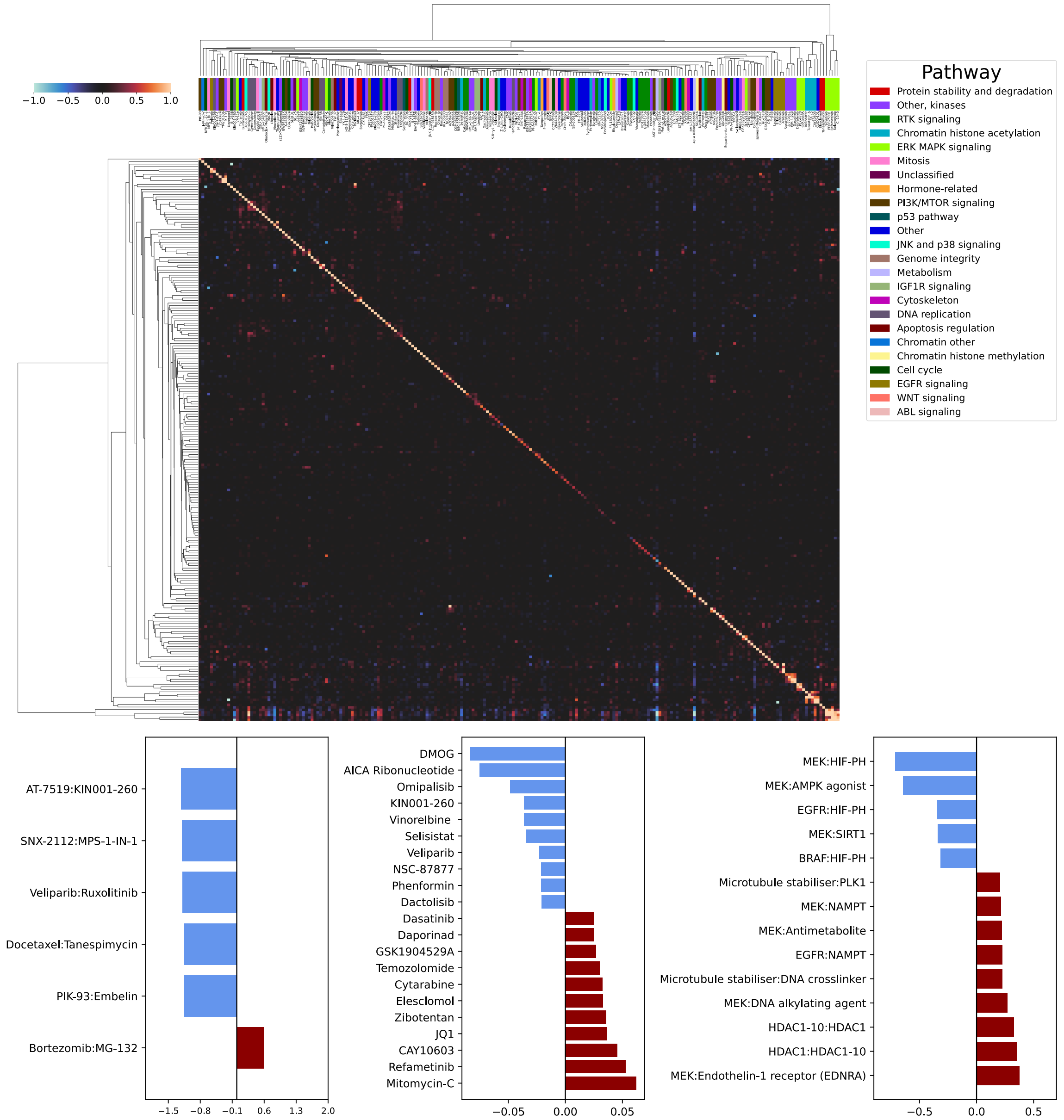

Fig E: **Coefficients of drugs present during training in XGDP-GNN.** One-to-one (bottom left), many-to-one (bottom middle), and class-to-class (bottom right) relationships are calculated from heatmap coefficients.

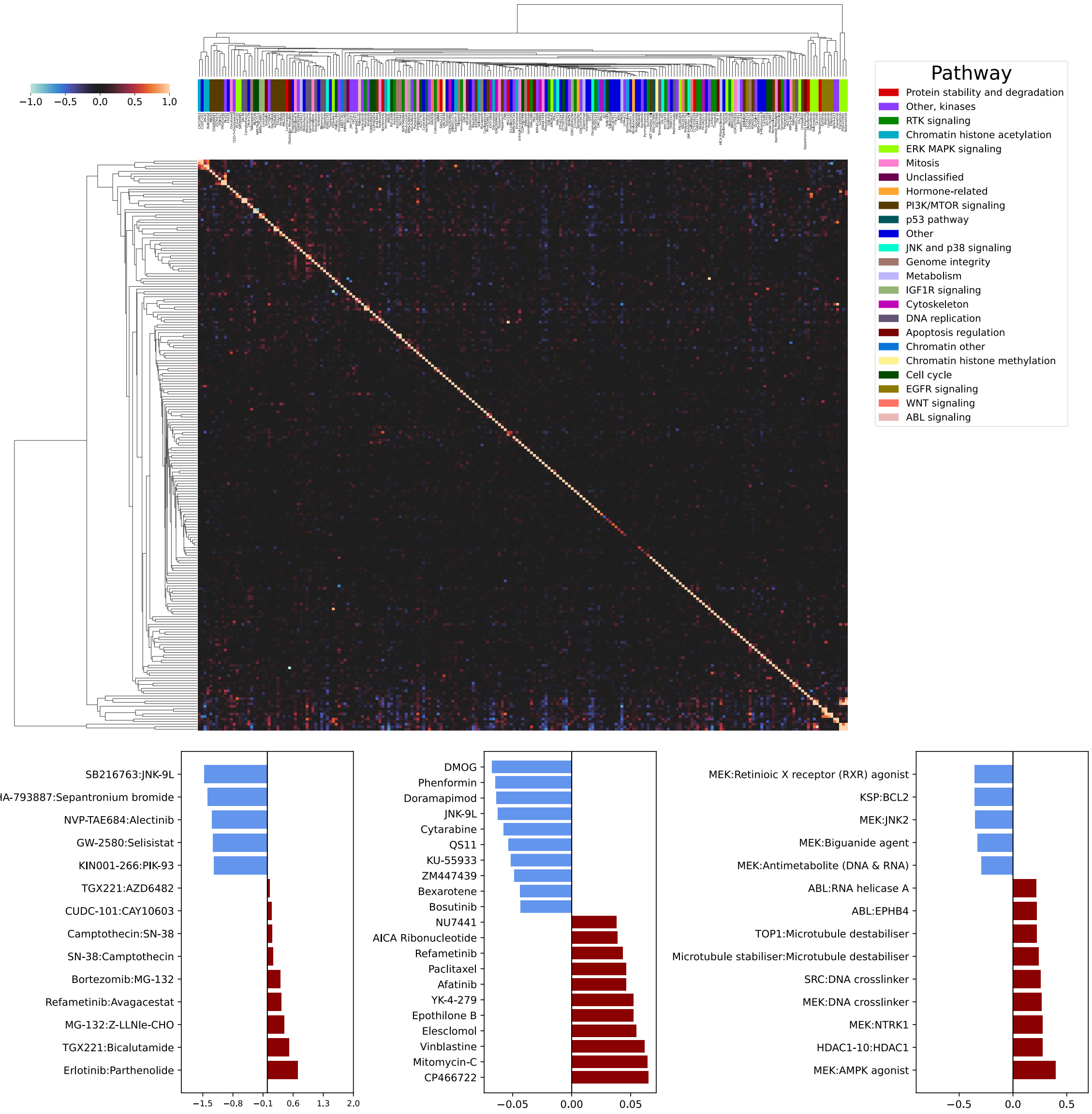

Fig F: **Coefficients of drugs present during training in XGDP-GAT.** One-to-one (bottom left), many-to-one (bottom middle), and class-to-class (bottom right) relationships are calculated from heatmap coefficients.

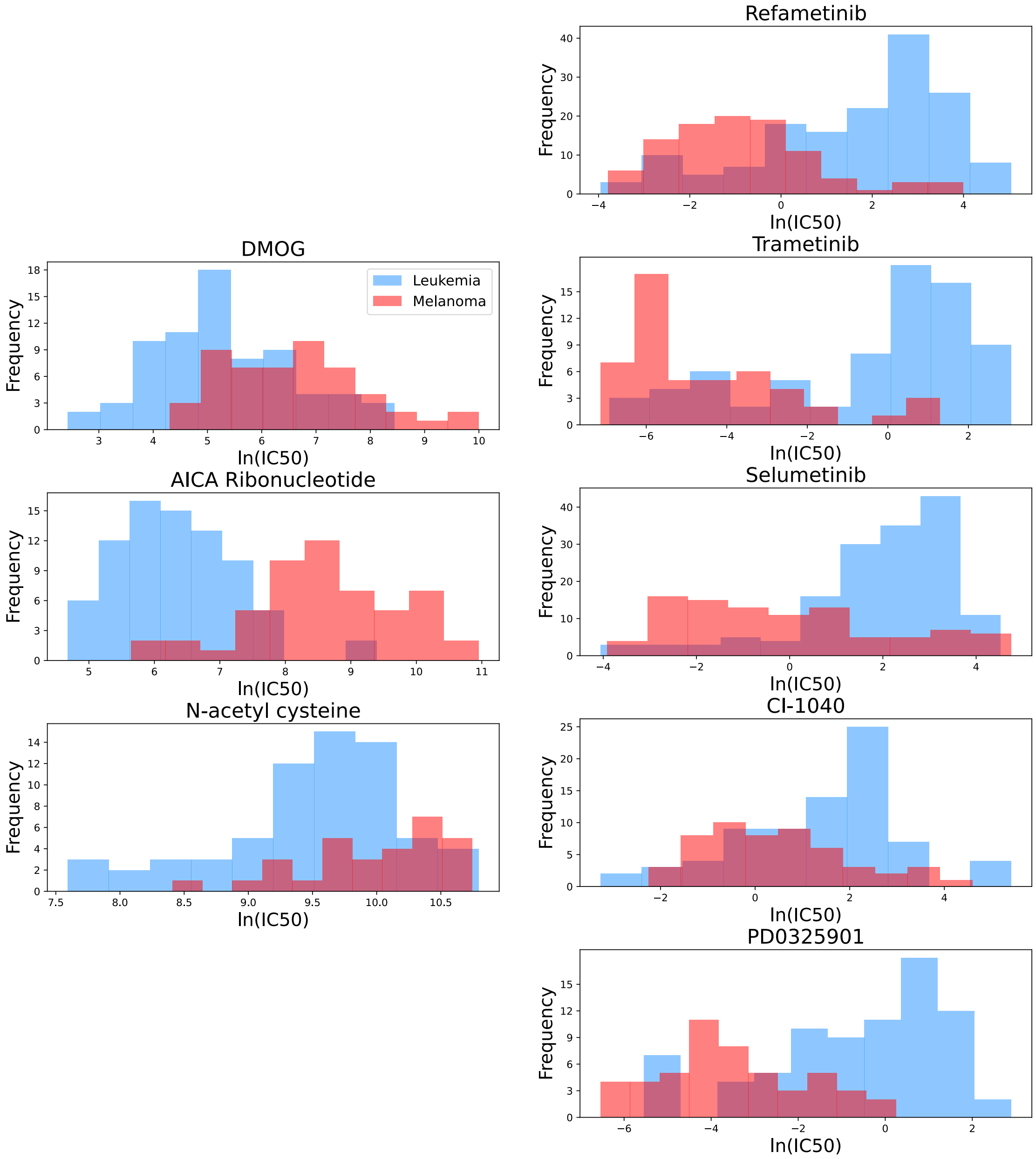

Fig G: **Metabolism targeting drugs (left) and MEK inhibitors (right) display distinct response profiles in leukemia and melanoma cell lines.** These plots display ln(IC50) values for leukemia (blue) and melanoma (red) cell lines in each respective drug (title of each plot). Ln(IC50) values are obtained from GDSC. Metabolism targeting drugs display higher relative efficacy for leukemia cell lines while MEK inhibitors have higher relative efficacy in melanoma cell lines.

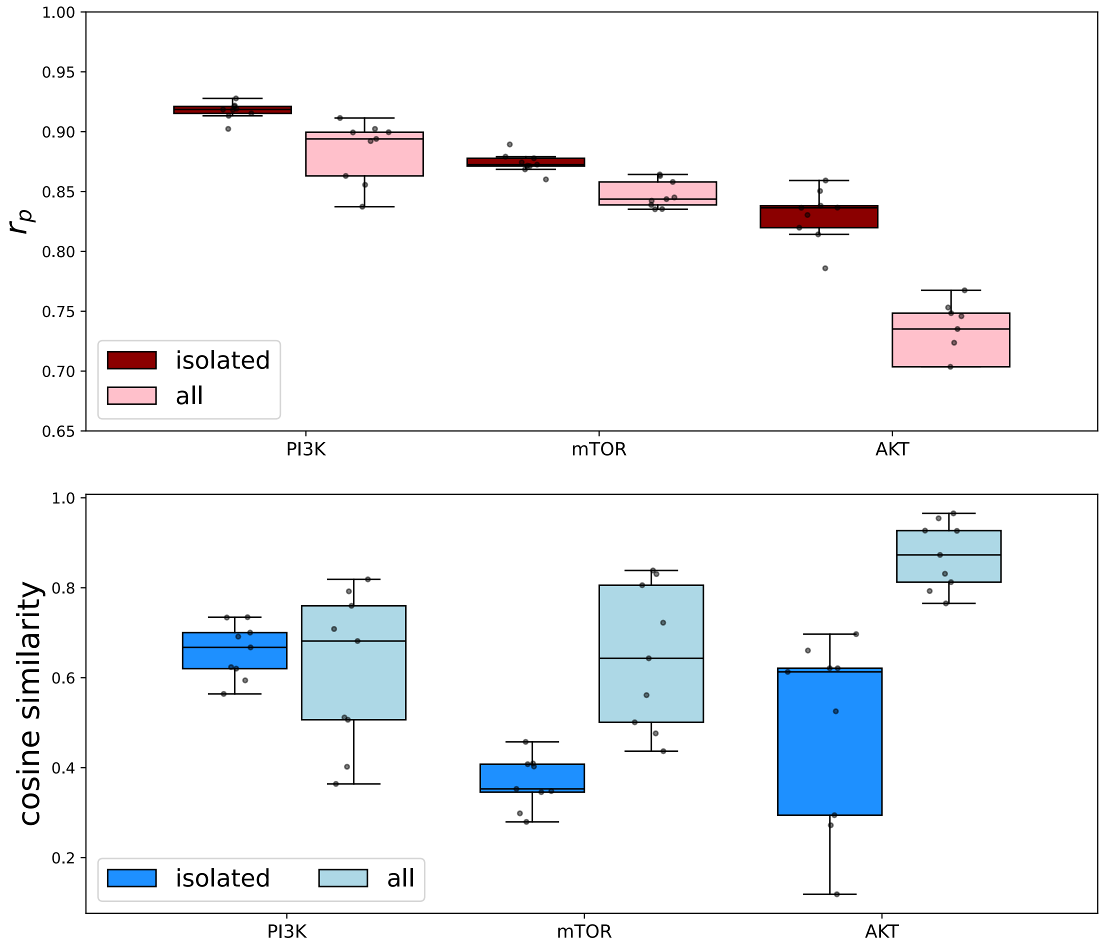

Fig H: **Training mechanism specific models on more specific targets for PI3K/mTOR pathway drugs improves performance and decreases intra-target drug representation similarity.**

Pathway	Mechanism	Drug count	Total samples	Pathway samples
PI3K/mTOR signaling	PI3K	10	5849	12453
	AKT	6	3693	
	mTOR	5	2911	
Mitosis	Microtubule inhibitor	4	2557	3856
	AURK	3	1419	
ERK MAPK Signaling	MEK	4	2557	6966
	BRAF	3	1900	
	ERK	3	1914	
	KRAS	1	595	
EGFR Signaling		7	4475	
DNA Replication	TOP1/2	6	3423	9978
	Alkylating agent	3	1922	
	Antimetabolite	2	1088	
	Pyrimidine inhibitor	2	1239	
	dsDNA break induction	1	501	
	G-quadruplex stabilizer	1	632	
	Anthracycline	1	637	
	DNA crosslinker	1	535	

Supplementary Table A. Counts of drugs and drug-cell pairings (total samples) used in mechanism specific model training.
