# Supplementary material for: Monotherapy cancer drug-blind response prediction is limited to intraclass generalization
Source: PLoS Comput Biol. 2026 Apr 22;22(4):e1013232. doi: 10.1371/journal.pcbi.1013232 (PMC13128123; doi:10.1371/journal.pcbi.1013232)
Supplement: Attachment — Submitted filename: response_to_reviewers.pdf [file pcbi.1013232.s002.pdf]

Response to Reviewers: Monotherapy cancer drug-blind response prediction is limited to intraclass generalization

William Herbert, Nicholas Chia, Paul Jensen, Marina Walther-Antonio

December 9, 2025

We thank all reviewers for their thoughtful and constructive comments. We appreciate your time and expertise to make this manuscript stronger.

Four general changes were made to the manuscript that were not requested by the reviewers:

1. The color scheme in figures is now colorblind friendly.
2. Figure 2B is replaced by an extended sensitivity analysis of specific mechanisms of action to a decreasing the number of cell line examples.
3. The mechanism-specific experiments are replicated (Figure 7). The intradrug permutation subfigure compares intradrug permutation in the whole dataset to mechanism-specific intradrug permutation. (Previously we compared normal mechanism-specific performance to mechanism-specific intradrug permutation.)
4. The mechanism families in Figure 5C now use the GDSC primary target annotations; all subsequent metrics in other models also use this style.

Please find our responses to comments from all reviewers below.

Comments from Reviewer 1:

> The current experimental validation relies exclusively on MLPs. Although the simplicity of this approach effectively isolates data-driven effects, it remains uncertain if these insights hold for more advanced architectures such as Graph Neural Networks or Transformers. Conducting an architectural ablation study, particularly on critical experiments such as permutation experiments or MoA-specific training, would significantly enhance the claim of architectural independence. Alternatively, referencing these results more explicitly from existing literature could serve a similar purpose.

We see the benefit of extending our experiments to other model architectures, especially since the field focuses on iterating new architectures using the same pharmacogenomic datasets. We repeated

the permutation experiments using a graph neural network (GraphCDR [1]) and a transformer-based cell line representation trained on single cell data (scFoundation [2]). The scFoundation model was particularly interesting since both the original scFoundation paper and other recent single-cell representation papers use cancer DRP as a benchmark [2, 3]. We examined GraphCDR as it binarizes drug response, which is a common preprocessing step in DRP papers. The accuracies of GraphCDR and scFoundation don't decrease after permuting the inputs. As shown in Supplemental Figure 1, the data leakages in our MLP model appear in other published architectures.

We repeated the heatmap experiment from Figure 4 using scFoundation embeddings as well as two different architectures (GNN and GAT) from XGDP [4]. (The GraphCDR model code could not be adapted to a partially drug-blind experimental setup.) The additional heatmap experiments allow us to make three types of comparisons:

1. **Same architecture, different cell line representation**: We tested raw gene expression representations against scFoundation cell line embeddings. While the non-drug-blind case remains largely unchanged, scFoundation embeddings cause a decrease in both intraclass information sharing as well as information sharing to drugs with broader or more promiscuous mechanisms of action, such as Lestaurtinib.

2. **Different sets of drugs during model training**: XGDP and our MLP experiment use semi-overlapping sets of drugs from GDSC1 and GDSC2 respectively. Intraclass information sharing requires a sufficient number of drugs with a shared mechanism.

3. **Two different model architectures trained on the same set of drugs**: We are able to directly compare GNNs and GATs in a drug-blind setting with the same set of drugs from GDSC1 within XGDP.

These results are shared in Supplemental Figures 4, 5, and 6 and described in a new results section 'Alternative cell line representation and model architectures impact drug-to-drug information sharing'.

> The intra-drug shuffle experiment convincingly shows models' biases towards learning average drug responses. Would it be plausible to conduct a complementary experiment where each drug's response values are replaced by a single representative value (like mean)? If the results are still similar to intra-drug shuffle, it would further confirm that the models are learning simplified drug-level heuristics.

A previous study used the average response of each drug as a prediction target [5] (reference 19 in our original submission). Branson et al. use this approach as a "drug average baseline" to compare DRP models. Another article by Li et al. ("Interpretable deep learning architectures for improving drug response prediction performance: myth or reality?" [6]) suggests using the drug average approach as the baseline for *all* DRP models rather than, for example, a Pearson correlation of zero. We note our agreement with this thinking and added references to the "average response" results of Branson et al. and Li et al. to the Results section ("Permutation of response values reveals dependence on drug distributions").

This comment motivated us to re-examine the predictions in our permutation experiments. The predicted response values tend to collapse to a constant — the mean of responses for a particular drug. These results are shown for nine selected drugs in Supplemental Figure 2. Our permutation

experiment mimicks a drug average baseline as outlined in the above studies. Even with incorrect cell line information, models perform relatively well because predicting the average response of a drug is not sufficiently penalizing. Overall, we conclude that drug average performance is the appropriate baseline for our study, and constant model outputs are also a good diagnostic indicator of mismatched or poor cell line information. (We found this behavior with the scFoundation model, and correspondence with the authors confirmed that all of the variability in panel B of Figure 3 in their paper is from "jitter added for visualization". Unfortunately, this concerning exchange raises questions about the validity of scFoundation's DRP results.)

> The analysis comparing mechanism-specific expert models to a global model effectively highlights performance gains from focused training. However, it is unclear whether improvements result from genuine specialization or from avoiding "negative transfer"/noise from unrelated drug classes in global training. This could be clarified by introducing another control, where a model is trained on the union of selected mechanism-specific subsets, but excluding unrelated drug classes.

The mechanism-specific classes shown in the expert models are an amalgamation of multiple related classes that can be subdivided. For example, we can partition PI3K/mTOR into PI3K drugs, mTOR drugs, and AKT drugs. However, Supplemental Table 1 illustrates that some classes are already too small for training, including some classes with only a single drug. We believe that the PI3K/mTOR split is the most appropriate class for testing the impact of splitting classes. Splitting PI3K/mTOR decreases drug-to-drug similarity for mTOR drugs and AKT drugs below the similarity of the entire class. The results of this test are shown in Supplemental Figure 6.

This comment touches on an important point for future DRP research: At what level should we be categorizing drug relationships? For example, further subsetting does not improve drug-to-drug similarity for PI3K drugs. PI3K targeting drugs could be further divided by isoform targets. We should prioritize data collection for drugs with a lack of diversity in drug representations to improve performance with a more aggressive splitting strategy. Larger, mechanism-specific datasets with more unique drugs would allow us to more carefully split pathways and subdivide drug classes.

We believe that both genuine specialization and lack of negative transfer improve DRP performance. It is encouraging that DRP improvement is coupled with decreased drug-to-drug similarity. Furthermore, intradrug shuffling performance does not change (Figure 7C). This shows our improvements is still relative to the drug-average benchmarks outlined above. It would concern us if intradrug shuffling in mechanism-specific models outperformed intradrug shuffling in the whole dataset, as this would indicate overfitting to individual drug distributions rather than pathway-level information. It seems that mechanism specific models perform better because they learn more separable drug representations, and we believe our revised Figure 7C better illustrates this point.

> The paper does an excellent job of revealing the fundamental limitations of current drug-blind evaluation paradigms, but it stops short of proposing actionable alternatives. The research community would benefit from clearly defined recommendations or a new standardized evaluation framework to guide future benchmarking efforts.

We've wrapped up the discussion by outlining four concrete recommendations:

1. DRP models should be tested against a drug average or intradrug permutation benchmark. This more accurately measures how much DRP models learn about cancer.

2. Traditional drug-blind benchmarking should be discontinued since it is too noisy. Improvement in a handful of drugs in three to five replicates is relatively meaningless. Training the required amount of models to determine a significant change in drug-blind performance allows someone to perform our more detailed drug-to-drug information sharing experiment anyways.

3. DRP model training should be stratified by drug MOA when possible. This is a more meaningful measure of model generalization, allows for more fine-grained architecture comparisons, and improves potential for clinical translation.

4. Future work should prioritize comparison of drug-to-drug information sharing in different response models than 2D culture, such as 3D organoids or PDXs in different organisms. This will improve our understanding of the complexity captured by these systems.

Comments from Reviewer 2:

> The use of a deep neural network with ten layers is difficult to reconcile with the study's focus on overfitting. Given the relatively limited number of samples, such a complex model increases the likelihood of overfitting, potentially undermining the main message of the paper. A more appropriate approach would have been to evaluate a range of simpler models to better support the analysis.

The overfitting we focus on in this study is a form of unintended data leakage. This is more of a data problem than an architecture problem. The main cause is that large pharmacogenomic datasets contain a relatively small set of unique drugs and cell lines. We also hope that the experiments performed in alternative deep learning architectures to address Reviewer 1's first comment also address some of the concerns of this comment.

The model does indeed begin to quantifiably overfit to individual cell-line pairings if trained for long enough. Test set performance begins to degrade while training set performance continues to improve. This is why we use early stopping with separate validation and testing sets. This approach is outlined in the Methods section ('Model architecture and optimization').

The behavior of simple models in cancer DRP has been well defined in the literature [7, 8]. Jang et al. in particular provide a thorough systematic analysis of these models. We are now in the second stage of the "double descent" of DRP models. Simpler models broadly underperform when compared to more advanced architectures, e.g. DNNs, transformers, GNNs, etc. [9]. We also hope that our response to Reviewer 3's comment asking for more detail on a citation in lines 21-23 of the revised paper also supports our architectural approach. Copied here, "Partin et al. find that deeper networks achieve improved performance over gradient boosting methods that is more appreciable as dataset size increases." The datasets used in this study are on the same scale we use here.

Drug-blind failure really only becomes noticeable in more advanced models because they have addressed the shortcomings of simpler models. This work is meant to diagnose and address drug-blind benchmarking in the context in which it is currently being implemented.

> The manuscript lacks readability in several places. Many sentences are overly long and complex, which affects the overall flow of the text. I recommend a thorough revision to improve clarity and ensure the manuscript reads more smoothly.

We have made an effort to break up complex sentences from the first draft. We hope this is appropriately reflected in the edits. Examples are in lines 2-8, 18-24, 101-103, 141-151, and 153-155.

> The message conveyed by Figure 4 is unclear. Additionally, Figure 4a is difficult to read due to its visual complexity.

The primary message conveyed by Figure 4 is multi-part, and we summarize the main components here as follows:

1. Non-drug-blind performance is far better than drug-blind performance. This can be seen by the extremely high coefficients along the diagonal.

2. Drugs with shared mechanisms of action do share information in the drug-blind setting. This is seen in the squares of shared coefficients highlighted in Figure 4b.

3. The majority of drug-to-drug relationships do not involve any information transfer. We can visualize this as the sparsity of coefficients in Figure 4, which requires the whole heatmap to appreciate.

We have updated the references of Figure 4 in the text to align with the statements corresponding to these components.

We believe that the actual graphic of Figure 4a should remain in its entirety in the main body of the paper. It communicates a large amount of information about the most important experiment in the paper. The figure is designed so that certain readers may find more specific information in it should they desire, especially if it is being viewed electronically. For example, we include the names of drugs in small font in case someone would like to zoom in and examine a column for a specific drug or set of drugs.

We recognize the complexity of Figure 4a, so we attempt to highlight different portions of it. We broke out sections of Figure 4a in to Figure 4b to reduce the burden on readers if that level of drug-specific information is not important to them. We further broke out information from Figure 4a in Figure 5 in by defining a set of heuristics to describe relationships among drugs. We have made it more explicit in the text that both Figure 4b and Figure 5 are derivatives of Figure 4a.

> The rationale behind using a linear model with drug features as input is not clearly explained. The implementation details are also vague. For example, on page 19, line 125, the authors initially mention using a linear model, but only in the following sentence is it clarified that the model is in fact a regularized linear model (elastic net). This key detail should be stated up front, and the motivation for using this approach should be better explained.

We have changed all references to a linear model to explicitly state that it is an elastic net model. We use the elastic net model to map the presence of a drug during training to the performance of a drug during testing. In our case, these were not "drug features" (i.e. features describing the chemical properties of a drug) but were the binary presence/absence of a drug in the training set. We have also expanded upon the broader technical benefits of choosing elastic net for feature selection in the Methods section.

> The choice of deep learning architecture is not clearly justified. In the model shown in Figure 8, the hyperparameter choices appear either arbitrary or specifically tuned to enhance test set performance. Specifically, the model uses a 7-layer architecture for the cell line wing and a 4-layer architecture for the drug wing, with custom neuron counts per layer. Although the choice of all these hyperparameters need to be justified, the manuscript states on page 17, line 353, that "the primary focus of

> this work was not to spend time identifying a hyperparameter tuning strategy." This is concerning, as hyperparameter tuning is a fundamental step in machine learning. Even simpler models such as the elastic net, with only two hyperparameters, require a tuning via nested cross-validation, which is also absent in this manuscript.

The specific layer dimensions are based off previously published two-arm MLP architectures used in cancer DRP. This has been made more explicit in the Methods. The relevant citations for these models are [10, 11]. The choice of two-arm approach over a concatenated input is also supported by the results of [10]. We have further clarified this in the Methods section.

The primary difference between these published models and ours is that we did not pre-filter gene expression features. We believe that preprocessing cell line features can bias models towards human interpretations of cancer, e.g. it is not appropriate to filter to only "cancer-relevant" genes. This required our cell line arm to be deeper than theirs. We removed the first two layers when performing the scFoundation experiments to address Reviewer 1's first comment as their cell line representation was already small enough and trained on a significant amount of single-cell data. We have described this in the Methods section.

The quote the reviewer points out was not intended to communicate that we spent no time at all identifying appropriate hyperparameters for the model. This quote was only intended to provide a distinction between architecture- and data-centric approaches and underline that in this paper we do not intend to compete for a model that is "state of the art". We have updated the language of this statement to reflect a more positive data-focused intention. Furthermore, all experiments and replicates in this paper are performed using random seeding of train/validation/test splits. We hope this clarifies that we have no motivation to be tuning hyperparameters to inflate our test set accuracy.

We did perform basic hyperparameter tuning. Batch size and learning rate were identified using a grid search strategy. This has been clarified in the Methods section. The elastic net parameters (alpha and l1_ratio) were also chosen using grid search and 10-fold cross-validation on random subsets of unique drugs. This has also been clarified in the methods, and elastic net training code for the heatmap has been added to the Github under the file analysis.ipynb.

> On page 8 line 133, there seems to be a redundant "be": "Therefore, drugs are be,".

The redundant "be" has been removed.

> Whenever a Python package is mentioned, its version should also be provided to ensure reproducibility.

Python package versions have been added for references to PubChemPy, RDKit, and scikit-learn in the Methods section.

The GitHub repository needs to be well documented and organized better.

A detailed README outlining the purpose and usage of each file in the Github has been added.

Comments from Reviewer 3:

> I think the authors would benefit from a conclusion section that emphasizes their core findings and discoveries, many of which are very interesting, especially related to the diversity of compounds in DRP datasets and the impact of this information on prediction, instead of the speculatory content provided.

We have modified our conclusion to center primarily around a review of core findings of this paper and their impact on the broader cancer DRP community.

> Could the authors consider why some drug groups in Figure 6 remain disparate even after model training? Specifically, the PI3K drugs (blue dots) are disperse in Figure 6A, but even at epoch 100, they aren't all cleanly grouped together, but seem to form distinct clusters within this mechanism of action. Again, this might highlight that even if drugs share at least one "primary target" that they could have distinct secondary or "off-targets".

This is an interesting question, and we thank the reviewer for their attention to detail. We have examined the distinct clusters of PI3K drugs in Figure 6C (Epoch 100), and they correspond to 3 different groups:

1. The closer group to EGFR and MEK/ERK is composed of all AKT inhibitors with some PI3K inhibitors.
2. The cluster closer to DNA replication and mitosis drugs is composed of a mixture of mTOR and PI3K inhibitors.
3. The PI3K drugs in the bottom right are the two PI3K inhibitors with strong negative information sharing discussed in the earlier results - AMG319 and CZC2832 - and LJI-308, an RSK inhibitor marked as PI3K/mTOR pathway by GDSC.

This underscores the further splitting performed in response to Reviewer 1's third comment. We can potentially use drug representations learned in the whole dataset model to decide how to train mechanism specific models. If a target has a well defined region in the drug representation feature space, it is possible to improve performance by focusing on that region. The dispersion of PI3K specific targets across the first two groups may also explain why training a mechanism specific model on PI3K inhibitors does not result in decreased drug-to-drug similarity.

> For the elastic net in Figure 3B, is there any way to also plot the "predicted" or "real" score against some metric of training set drug "uniqueness"

A potential measure of training set "uniqueness" would be to determine how many different mechanisms of action are present in each different training set. These results are shown in Supplemental Figure 3. We find that there is no clear correlation of training set uniqueness to performance when measured at the pathway level or at the specific target level. This further strengthens the examination of individual drug-to-drug relationships that follows.

> In the results, it's a big jump to the permutation testing. What dataset was used and can you give a rough sense of scale of how many drug-cell line combinations were considered and how many drug repeats were used? It's hard to understand how you did intra-drug shuffling without know how many or the nature (same dose?) of repeat drug samples. Similarly in the methods, is there a count of all drugs obtained and their replicates, if available?

The dataset used for permutation testing is denoted in parentheses in Figure 1. We have emphasized this further in the figure caption. We have added the full number of combinations for each dataset to the Methods section. We are not certain what the reviewer means by "repeats". If they are referring to technical replicates, this depends on the dataset. GDSC calculates IC50 by pooling across all replicates (`https://www.cancerrxgene.org/help#t_curve`). If the reviewer is referring to repeated cell-drug pairings, there are 6288 of these in the raw GDSC dataset, or 2.5% of the total number of experiments. They also tend to be concentrated in a small amount of drug/cell pairings. It would be rare for a response to be accidentally "shuffled" to the same cell-drug pairing.

> Also, is there a way to a priori characterize their mechanisms of action since the introduction so heavily emphasizes the importance of this information?

Mechanism of action as reported in this paper is taken directly from the mechanisms of action GDSC (and other datasets) define for each drug. GDSC in particular provides two levels of mechanism annotation: 'Targets' and 'Target pathway'. We use the broader 'Target pathway' for annotating heatmap categories. We use 'Targets' in Figure 5C and in experiments performed to address Reviewer 1's third comment.

> Was there any overlap in the drugs or cell lines? And how were the response data harmonized across the three sources? Or did they all report the same response?

Overlap and consistency of responses reported by large pharmacogenomic datasets is an important topic, and one that we reference in the introduction to this paper [12]. In our experiments, the three sources of data were never pooled together. They were only examined individually. Therefore, we did not harmonize across them.

I very much appreciate the discussion of the shared coefficients from Figure 4 – it's interesting when drug targets in the same pathway can inform the model's ability to predict unseen drugs. It would be helpful to describe how this information was obtained? The Figure 4 caption suggests that it was provided by GDSC, but the authors only mention drug fingerprints in the method and give no indication of retrieving their MOA or how these annotations were discovered.

We are glad you enjoyed this part of the paper, as we think it's the most eye-opening experiment! We hope that the information provided above regarding how mechanisms were defined helps address this comment. We have also added a section in the methods specifically addressing the location of the mechanisms within the data obtained from GDSC.

The GitHub respository is not public and so I cannot verify that the code and data are available.

The GitHub repository was made public and successfully accessed by the two other reviewers. I have reviewed the permissions on the GitHub and had coworkers attempt to access the public link with success as well. Please let me know if you are still unable to access the link, as we care deeply about the transparency of our code.

Is next-in-class prediction entirely predictable for drug-blind tasks? The authors show that this depends greatly on the drug MOA as some classes, this is less true. Similarly, the authors also previously alluded to the fact that drugs with similar MOAs did not always have high fingerprint similarity. The last paragraph of the discussion is the most interesting in the context of this study's results, but seemingly contradicts this earlier paragraph.

Next-in-class prediction and predicting drugs with promiscuous mechanisms of action (the last paragraph of the discussion referred to) are separate tasks. Next-in-class prediction would be applied to drugs designed for a particular purpose, such as another PI3Kα inhibitor. Identification of promiscuous drugs would be applied to identify potential off-target uses. Drugs identified using next-in-class prediction could be found to be promiscuous, absolutely, but we do not believe the existence of promiscuous drugs prevents us from predicting next-in-class efficacy.

Generally, the discussion would benefit from a first paragraph that summarizes the authors goals and findings before jumping to the conclusion that models only predict MOA

Two paragraphs reviewing our goals and results have been added to the beginning of the discussion.

> I wonder if the authors would consider highlighting a few concrete findings from these papers into these introduction sentences, which are intriguing: Early work examined the general scaling laws applicable to these datasets [8]. This is important for guiding further collection and experimentation. Different datasets also display different levels of efficacy for prediction among one another, showing that not all cancer DRP datasets are created equal." What was learned about the general scaling? Was there a quantification of the extent of prediction from one dataset to another? These would be good numbers to contextualize the authors results in figure 6 & 7.

We have added more details to contextualize the importance of these references to our paper. They are shown on lines 27-29 and 31-35 in the tracked changes.

> Could the authors better define dataset diversity? If the mechanism of action is so important, is there a way to first quantify the "diversity" of MoA? Perhaps even by clustering all of the fingerprints to show that each dataset has X unique groups? And then we selecting the test set, how was the "uniqueness" of the test set determined? This would be different than clustering drugs with annotated MOAs like in Figure 6.

Dataset diversity refers to the number of cell lines a drug is tested on where it is used in the results section 'Quantifying the impact of dataset diversity on performance'. We have added a sentence clarifying this.

We do not explicitly choose any test sets in this paper based on a predefined diversity heuristic. In our response to one of your previous comments, we quantified 'uniqueness' of mechanisms of action in the training set. This is unrelated to the above definition of dataset diversity.

> The relationship between PD0325901 and PD173074 is interesting – I wonder if the authors may consider that their approach could uncover unknown drug targets when predictive utility is found between two compounds?

We do believe that this is a potential use case for the drug-to-drug information sharing measurement we outline in this paper, but at this point we can only speculate based on previous literature. It would be interesting to use this approach to mark certain drugs for further experimentation in a high-throughput data collection setting, such as an automated lab.

Figure 6 is interesting in that it concretely identifies similarity in terms of reported MOA. Could you describe this data in the methods section- were all compounds annotated this way? In the results it says "selected compounds" but how many is that?

We added a further description of how MOA was obtained in the Methods in response to a previous comment. All drugs were provided with target information, even if it was the category "Other". The number of drugs and pairs of the selected mechanisms were added in Supplemental Table 1 to address a previous reviewer comment.

Please define all x-axis terms in the Figure 1 caption.

All drug response metrics from Figure 1 have been defined in the caption.

It would be helpful to make font sizes on figures larger throughout. Especially Figure 6 and 7.

Font sizes on figures have been increased throughout the paper.

In figure 8, the caption doesn't sufficiently describe why there are multiple rectangles with decreasing numbers. Is this meant to show differences in features per cell line or that there is some prioritization of features before predicting the response?

This is meant to show the decreasing number of features at each layer of the MLP. Each successive layer decreases the total number of features used to represent the cell line or drug as a function of previous features. We have added a sentence to the figure caption further explaining this.

Does "mechanism of action" always refer to documented drug targets or something else? This is largely discussed for GDSC data, but do the other datasets contain any MOA information?

As we refer to them in this paper, mechanism of action refers to documented drug targets reported by the dataset from which responses are obtained. Other datasets do contain MOA information. For CTRP, one can navigate to the DepMap portal and download a manifest of compounds (`https://`

depmap.org/portal/data_page/?tab=allData). PortalCompounds.csv contains columns 'TargetOrMechanism' as well as 'GeneSymbolOfTargets'. When examining MOA, we think it is most appropriate to use the annotations provided by the source of the response dataset for the sake of consistency. Still, it is important to consider target promiscuity and dataset consistency when using these annotations. For example, at the broader level, Lestaurtinib is labelled 'Other, kinases' in GDSC and 'FLT3 INHIBITOR, GROWTH FACTOR RECEPTOR INHIBITOR, JAK INHIBITOR' in DepMap.

> In one of the only efforts to directly address drug-blind failure, performance was improved using multi-objective optimization to prevent training from skewing performance to specific drugs by enhancing the extraction of global drug response features" Could the authors more concretely describe "global drug response" features? This comes up later and would be good to define.

In this case, the referenced study focuses specifically on an optimization algorithm that prioritizes drug-blind performance. This is likely at the cost of individual drug performance, which would be more 'local' features. The reference contains an interesting figure describing this as a Pareto front. We have included this definition where this paper is referenced on lines 66-68 in the tracked changes.

> The relatively high performance even with a low number of input samples is interesting, and has been shown in other places in the literature as well. I wonder if this paper, specifically figure 4C would be of interest to the authors. Dataset size is a very important topic for ML.
>
> Yuan B, Shen C, Luna A, Korkut A, Marks DS, Ingraham J, Sander C. CellBox: Interpretable Machine Learning for Perturbation Biology with Application to the Design of Cancer Combination Therapy. Cell Syst. 2021 Feb 17;12(2):128-140.e4. doi: 10.1016/j.cels.2020.11.013. Epub 2020 Dec 28. PMID: 33373583.

We appreciate the reviewer pointing our attention towards this paper. We agree that the gains in performance while using far less data are surprising, but we believe that it mostly underscores the lack of information sharing among drugs. Rather than training on one large drug response dataset, it's as though this model is just learning from many small datasets separately.

> Given the differences in the dataset size, prediction may be influenced by the response score and response score distribution. Could the authors give any insights about the differences in the response variables?

While we use gCSI specifically to examine different response variables, we recognize it is the smallest of the three datasets we test. This is the primary bottleneck to wider adoption of the gCSI

dataset. The creators of gCSI postulate that GR based response metrics correct for cellular division time which confounds EC50 measurements [13].

Response variables are largely normally distributed in the three datasets, but the same response variable can be reported differently. For example, EC50 in GDSC is reported as ln(EC50), while in CTRP it is reported as 'apparent' EC50 μM.

We gain coverage over different response metrics (growth rate and cell viability) by using gCSI. By using both GDSC and CTRP, we cover reporting differences as well. Furthermore, it is known that there are inconsistencies in large pharmacogenomic datasets, so we are also controlling for different site to site technical variation as well.

> For gCSI, can you give a range of cell lines per drug?

A range of unique cell lines in gCSI has been added to the Methods section.

> For the tuples, how many features were extracted for cell lines? What is the intersection size with the TCGA data? Again, it's likely sufficient to give a range. Also were all cell lines from the three DRP datasets available in TCGA?

The number of gene expression features can be seen in the first layer of the model architecture in Figure 8. There are 19194 genes in the cell line input. Intersecting with TCGA gene expression features removed around 80 genes. TCGA contains expression data from patient tumors only, not cell line data. This intersection was done for planned future work with patient data so that pretrained models from experiments in this paper could be used with patient gene expression data from TCGA.

> Could the authors give a more concrete definition of composition in the "Prediction of performance by training set composition" as well?

Composition in this case refers to the binary presence/absence of a particular drug in the training set. We have clarified this in the Methods section.

> In the results and methods section, can the authors provide more detail about how they did the one-hot encoding?

We borrow the term one-hot encoding from the artificial intelligence field [14]. One-hot encoding is a way to convert categorical data in to a vector representation. For example, if each unique cell line is a "category", we have an input vector that is the length of the number of unique cell lines. Rather than using the gene expression of the cells, we use a vector where the index corresponding

to that unique cell line is 1 while all other values are 0. This allows us to featurize an input without providing any further knowledge than its presence. This then allows us to identify the benefit of any further information relative to the one-hot condition.

> In the results section it would be helpful to remind the reader which drug response datasets are being used for each analysis, knowing that not all datasets had sufficient information for each analysis.

We have added in a reference in the results section denoting where all further experiments are done only with GDSC.

References

[1] Liu X, Song C, Huang F, Fu H, Xiao W, Zhang W. GraphCDR: a graph neural network method with contrastive learning for cancer drug response prediction. Briefings in Bioinformatics. 2022;23(1):bbab457.

[2] Hao M, Gong J, Zeng X, Liu C, Guo Y, Cheng X, et al. Large-scale foundation model on single-cell transcriptomics. Nature methods. 2024;21(8):1481–1491.

[3] Theus A, Barkmann F, Wissel D, Boeva V. CancerFoundation: A single-cell RNA sequencing foundation model to decipher drug resistance in cancer. bioRxiv. 2024; p. 2024–11.

[4] Wang C, Kumar GA, Rajapakse JC. Drug discovery and mechanism prediction with explainable graph neural networks. Scientific Reports. 2025;15(1):179.

[5] Branson N, Cutillas PR, Bessant C. Understanding the sources of performance in deep drug response models reveals insights and improvements. Bioinformatics. 2025;41(Supplement_1):i142–i149.

[6] Li Y, Hostallero DE, Emad A. Interpretable deep learning architectures for improving drug response prediction performance: myth or reality? Bioinformatics. 2023;39(6):btad390.

[7] Jang IS, Neto EC, Guinney J, Friend SH, Margolin AA. Systematic assessment of analytical methods for drug sensitivity prediction from cancer cell line data. In: Biocomputing 2014. World Scientific; 2014. p. 63–74.

[8] Park A, Lee Y, Nam S. A performance evaluation of drug response prediction models for individual drugs. Scientific Reports. 2023;13(1):11911.

[9] Partin A, Brettin TS, Zhu Y, Narykov O, Clyde A, Overbeek J, et al. Deep learning methods for drug response prediction in cancer: predominant and emerging trends. Frontiers in medicine. 2023;10:1086097.

[10] Partin A, Brettin T, Evrard YA, Zhu Y, Yoo H, Xia F, et al. Learning curves for drug response prediction in cancer cell lines. BMC bioinformatics. 2021;22:1–18.

[11] Zhu Y, Brettin T, Evrard YA, Partin A, Xia F, Shukla M, et al. Ensemble transfer learning for the prediction of anti-cancer drug response. Scientific reports. 2020;10(1):18040.

[12] Haibe-Kains B, El-Hachem N, Birkbak NJ, Jin AC, Beck AH, Aerts HJ, et al. Inconsistency in large pharmacogenomic studies. Nature. 2013;504(7480):389–393.

[13] Hafner M, Niepel M, Chung M, Sorger PK. Growth rate inhibition metrics correct for confounders in measuring sensitivity to cancer drugs. Nature methods. 2016;13(6):521–527.

[14] Russell SJ, Norvig P. Artificial Intelligence: A Modern Approach. 4th ed. Pearson; 2020.
