# Supplementary material for: Monotherapy cancer drug-blind response prediction is limited to intraclass generalization
Source: PLoS Comput Biol. 2026 Apr 22;22(4):e1013232. doi: 10.1371/journal.pcbi.1013232 (PMC13128123; doi:10.1371/journal.pcbi.1013232)
Supplement: Attachment — Submitted filename: drug_blind_generalization_paper_response_2.pdf [file pcbi.1013232.s003.pdf]

Response to Reviewers: Monotherapy cancer drug-blind response prediction is limited to intraclass generalization

William Herbert, Nicholas Chia, Paul Jensen, Marina Walther-Antonio

February 26, 2026

Comments from Reviewer 2:

> While the additional experiments with GraphCDR and scFoundation support the argument that increasing architectural complexity does not resolve drug-blind generalization, it is still not fully established that the observed failure mode is independent of model architecture. In addition, the manuscript still does not demonstrate whether simpler models exhibit the same qualitative behaviors that underpin the paper's conclusions. The motivation for requesting simpler baselines was not to improve performance, but to more cleanly diagnose the nature of the learnable signal.
>
> Regarding the justification of the two-arm MLP architecture, I appreciate the addition of references [10, 11]. Having reviewed these works, I agree that they employ conceptually similar two-branch designs for drug response prediction. However, the architectures are not identical in depth, width, or layer-wise configuration, and the specific asymmetry adopted here (e.g., a deeper cell-line arm with custom layer sizes) remains only loosely motivated by precedent. Given the manuscript's explicit data-centric framing, the absence of architectural sensitivity or ablation analyses leaves some uncertainty as to how much these design choices influence the observed behaviors.
>
> While I appreciate the clarification that batch size and learning rate were selected via grid search, these parameters primarily affect optimization process rather than architectural capacity or inductive bias. As such, they do not directly address concerns related to model complexity, and representational power. The core architectural hyperparameters (e.g., number of layers, layer widths, and asymmetry between model arms) remain fixed and largely unexplored. The distinction between optimization hyperparameters and architectural hyperparameters is important.

We have grouped these comments together as they all center around the value of examining alternative versions of our baseline MLP model. Using the permutation experiment as our metric, we performed experiments using a variety of different layer configurations and included this below. The cell embed, drug embed, and calc. response columns display the consecutive layer sizes in each corresponding component of the model architecture shown in Figure 8 in the main paper.

Cell embed	Drug embed	Calc. response	Normal	Intradrug	Intracell	Intradrug/Normal
[1000]	[100]	[50, 25]	0.9307	0.8407	0.3084	0.90331
[1000]	[100, 50]	[50, 25]	0.9276	0.8408	0.3122	0.90644
[1000]	[100, 50, 25]	[50, 25]	0.9221	0.8403	0.3139	0.91128
[1000, 750]	[100]	[50, 25]	0.9335	0.8404	0.3117	0.90028
[1000, 750]	[100, 50]	[50, 25]	0.9317	0.8420	0.3148	0.90372
[1000, 750]	[100, 50]	[250, 100, 25]	0.9331	0.8419	0.3131	0.90229
[1000, 750]	[100, 50, 25]	[50, 25]	0.9266	0.8408	0.3141	0.90736
[1000, 750, 500]	[100]	[50, 25]	0.9332	0.8419	0.3116	0.90218
[1000, 750, 500]	[100]	[250, 100, 25]	0.9350	0.8418	0.3112	0.90038
[1000, 750, 500]	[100, 50]	[50, 25]	0.9319	0.8420	0.3139	0.90353
[1000, 750, 500]	[100, 50, 25]	[50, 25]	0.9299	0.8421	0.3127	0.90561
[1000, 750, 500, 250]	[100]	[50, 25]	0.9330	0.8417	0.3112	0.90219
[1000, 750, 500, 250]	[100, 50]	[50, 25]	0.9314	0.8418	0.3130	0.90384
[1000, 750, 500, 250]	[100, 50, 25]	[50, 25]	0.9310	0.8417	0.3129	0.90405
[1000, 750, 500, 250, 100]	[100]	[50, 25]	0.9305	0.8418	0.3106	0.90476
[1000, 750, 500, 250, 100]	[100, 50]	[50, 25]	0.9301	0.8419	0.3105	0.90513
[1000, 750, 500, 250, 100]	[100, 50, 25]	[50, 25]	0.9273	0.8413	0.3133	0.90726
[1000, 750, 500, 250, 100, 50]	[100]	[50, 25]	0.9276	0.8420	0.3088	0.90775
[1000, 750, 500, 250, 100, 50]	[100, 50]	[50, 25]	0.9290	0.8412	0.3095	0.90557
[1000, 750, 500, 250, 100, 50]	[100, 50, 25]	[50, 25]	0.9182	0.8418	0.3117	0.91675
NA	NA	[125]	0.9090	0.8393	0.3148	0.92329
NA	NA	[250, 125]	0.9220	0.8392	0.3145	0.91024
NA	NA	[500, 250, 125]	0.9221	0.8381	0.3158	0.90888
NA	NA	[1000, 500, 250, 125]	0.9250	0.8379	0.3147	0.90591

We observe the same pattern of performance drops in normal, intradrug, and intracell permutations across all 24 layer configurations. Given this, we do not plan to test these layer configurations using our drug-blind method.

> The clarification that the dominant form of overfitting studied here is data-driven is well taken and is supported by the permutation analyses. However, the strength of the manuscript's conclusions — particularly statements suggesting that drug-blind performance reflects dataset behavior rather than model behavior — would be better justified by showing that these phenomena persist across a broader spectrum of model capacity and drug features.
>
> In summary, while the evidence convincingly shows that current monotherapy pharmacogenomic datasets impose severe limits on drug-blind generalization, the stronger claim that this failure should be attributed primarily to data rather than model choice extends beyond what is directly demonstrated. If the authors do not intend to evaluate a substantially broader range of methods and feature representations—which would likely be outside the scope of the present work—then softening this claim, or more explicitly delimiting it to the architectures and settings used in the current study, would better align the conclusions with the evidence presented.

We have added language recognizing that, while we have established a strong foundation for defining data-driven failure in a drug-blind setting, true drug-blind performance is likely a function of both model architecture and the data. We confine our claims of data-driven performance to the fact that, in this present study, we were unable to identify a model in which drug-blind failure is not data-driven. Examples of these changes can be found on lines 74-78, 89, 357-358, 368, 378, 381, and 416 of the new revision with tracked changes. We agree with the reviewer that examination of a larger set of models is outside of the scope of this paper, and have tied it in to our recommendations on line 456.

> I appreciate the authors' effort to add a more detailed README to the GitHub repository. I encourage the authors to further improve reproducibility by clearly structuring the codebase and clarifying the recommended execution order. In addition, documenting the computational environment (e.g., Python version and package dependencies via a 'requirements.txt' or conda environment file) would help ensure the results can be reliably reproduced.

We have added a couple sentences at the beginning of the README more explicitly delimiting running order. Running order primarily applies to the need to do data preprocessing before experimentation. All experiments in this paper (permutations, heatmap, mechanism specific training) can be run independently following this. We have also added an environment.yaml file.
